# Hobday’s Canine and Feline Surgery

**Published:** 1900-11

**Authors:** 


### BOOK REVIEW.

Hobday's Canine and Feline Surgery.

This work, as the preface states, has been placed before the student and practitioner with a wish that it may prove of benefit to them, and to cover a field of special surgery (canine and feline) not as yet attempted.

The first portion of the work is devoted to the securing and preparation of the animal for operation, and the author shows a perfect familiarity with the subject, going minutely into the different parts of the manual with the force and detail of a master of the subject on which he writes.

The descriptions are lucid, and cannot but be of great benefit to the student and also to the practitioner. We cannot but feel, however, that while the different methods are all very well for a hospital with its corps of assistants, its various apparatus, etc., it is a question whether the average practitioner and young graduate can only sigh for the possession of such luxuries, and confine himself to the simpler methods of restraint.

The continued use also of chloroform in anæsthesia, when there is such a drug as ether, is a matter of constant wonder to practitioners on "this side of the water." We have tried time and again to use the former drug, and did it with fear and trembling, and while the percentage of sudden collapse has not been large, still it has been sufficient to wean us from it and to take a drug like ether, and there have not only some latitude but lots of it; where we can be our own assistant and give our own ether or instruct a layman to assist, and feel that we can go on with our operation with safety; where one can depend on himself, for it is not always convenient, or is there enough pecuniary return to share the remuneration with a trained assistant?

The operations, as the author states in his preface, are described in a concise manner, and we are inclined to agree with the author that the words have been followed out to the letter; but we think he has been too concise in his descriptions, for there are a number of instances where it would puzzle the student or practitioner to perform the operation unless he had previously seen it performed, or was guided by some one who was proficient in it.

The text is extremely simple, and the grammar, which to our mind is good, would commend it to certain reviewers who fail to look on a work for its intrinsic value, but go into holy horror over a grammatical error.

The photographic half-tones are very good, and the author deserves great credit for originality. The balance of the illustrations (special apparatus, surgical apparatus) would look much better in a surgical instrument maker's catalogue than in a scientific work. There has been recently, and, in fact, always to our mind, too much placing of cuts of various instruments and surgical apparatus in text-books where the maker's name was boldly displayed, giving the work the air of an advertising dodge.

The work, however, is one that should commend itself to the profession and to the student, and, to copy a thread-worn expression, "it fills a long-felt want," containing as it does a description of all the operations performed on the dog and cat in a clear, simple way, and with it a number of original ideas, especially as to the apparatus, which are very valuable, and for which the author deserves praise from every member of his profession, especially those interested in the small animals.

A. G.

---

The well-known firm of Messrs. E. R. Squibb & Son are now making a feature of their work the preparation of private formulæ, so that nicety of product, perfection of combination, and attractive appearance that an ordinary pharmacy will not permit of will be at the command of the veterinary profession.

Veterinary sanitary work in the City of Manila, P. I., has already received much attention through our army officials stationed there, and food-inspection, especially of pork-products, becomes a very needful field of work, owing to the frequency of tapeworm among the natives.

